# Do Patients Thought to Lack Consciousness Retain the Capacity for Internal as Well as External Awareness?

**DOI:** 10.3389/fneur.2018.00492

**Published:** 2018-06-27

**Authors:** Amelie Haugg, Rhodri Cusack, Laura E. Gonzalez-Lara, Bettina Sorger, Adrian M. Owen, Lorina Naci

**Affiliations:** ^1^Department of Psychiatry, Psychotherapy and Psychosomatics, Psychiatric Hospital, University of Zurich, Zurich, Switzerland; ^2^Neuroscience Center Zurich, Swiss Federal Institute of Technology, University of Zurich, Zurich, Switzerland; ^3^School of Psychology, Trinity College Institute of Neuroscience, Trinity College Dublin, Dublin, Ireland; ^4^The Brain and Mind Institute, University of Western Ontario, London, ON, Canada; ^5^Faculty of Psychology and Neuroscience, Maastricht University, Maastricht, Netherlands

**Keywords:** functional Magnetic Resonance Imaging (fMRI), functional connectivity, disorders of consciousness, naturalistic stimulation, movie watching, conscious information processing

## Abstract

It is well established that some patients, who are deemed to have disorders of consciousness, remain entirely behaviorally non-responsive and are diagnosed as being in a vegetative state, yet can nevertheless demonstrate covert awareness of their external environment by modulating their brain activity, a phenomenon known as cognitive-motor dissociation. However, the extent to which these patients retain internal awareness remains unknown. To investigate the potential for internal and external awareness in patients with chronic disorders of consciousness (DoC), we asked whether the pattern of juxtaposition between the functional time-courses of the default mode (DMN) and fronto-parietal networks, shown in healthy individuals to mediate the naturally occurring dominance switching between internal and external aspects of consciousness, was present in these patients. We used a highly engaging movie by Alfred Hitchcock to drive the recruitment of the fronto-parietal networks, including the dorsal attention (DAN) and executive control (ECN) networks, and their maximal juxtaposition to the DMN in response to the complex stimulus, relative to rest and a scrambled, meaningless movie baseline condition. We tested a control group of healthy participants (*N* = 13/12) and two groups of patients with disorders of consciousness, one comprised of patients who demonstrated independent, neuroimaging-based evidence of covert external awareness (*N* = 8), and the other of those who did not (*N* = 8). Similarly to the healthy controls, only the group of patients with overt and, critically, covert external awareness showed significantly heightened differentiation between the DMN and the DAN in response to movie viewing relative to their resting state time-courses, which was driven by the movie's narrative. This result suggested the presence of functional integrity in the DMN and fronto-parietal networks and their relationship to one another in patients with covert external awareness. Similar to the effect in healthy controls, these networks became more strongly juxtaposed to one another in response to movie viewing relative to the baseline conditions, suggesting the *potential* for internal and external awareness during complex stimulus processing. Furthermore, our results suggest that naturalistic paradigms can dissociate between groups of DoC patients with and without covert awareness based on the functional integrity of brain networks.

## Introduction

In the last decade, a population of patients has been identified who are demonstrably conscious, but entirely unable to speak or move willfully in any way, and remain behaviorally non-responsive for several years (1–8). Following severe brain-injury, patients may manifest a spectrum of behavioral non-responsivity, from a complete absence to minimal and inconsistent willful behavioral responses (9–11). Patients who do not show any willful behavioral responses on repeated behavioral examinations, are thought to lack awareness of oneself and one's environment and are clinically diagnosed to be in a vegetative state (VS) (12), also known as the “unresponsive wakefulness syndrome” (UWS) (13). The clinical, behavioral assessment of non-responsive patients is particularly difficult because of its reliance on the subjective interpretation of inconsistent behaviors, which are often limited by motor constraints, and can result in high misdiagnosis rates (up to 43%) (14). Recent studies have shown that, despite the complete absence of external signs of awareness, a significant minority (~14–19%) of patients thought to be in a VS are able to demonstrate conscious awareness by modulating their brain activity (2, 15) in different types of neuroimaging paradigms [e.g., (1, 3–5, 7, 8)], a phenomenon captured by the recently-coined term “cognitive motor dissociation” (CMD) (16). Despite these advances, the mental life of behaviorally non-responsive patients—particularly their capacity to have similar experiences as healthy individuals in response to everyday life events that involve both their *awareness of oneself* and *awareness of one's environment*—had until recently remained largely unknown and inaccessible to empirical investigation.

To address this challenge, Naci et al. (7, 17, 18) developed a movie-viewing paradigm for the investigation of conscious experiences of behaviorally non-responsive patients who may retain covert awareness. Movie viewing is highly suited to testing populations that exhibit large fluctuations in arousal, impaired motor control and compromised attention span, because, by creating an immersive experience, it naturally engages attention and various cognitive processes that lead to reduced movement in the scanner (19) and recruitment of strong brain activity that is synchronized across different individuals (7, 18, 20–23). Naci and colleagues focused on the assessment of executive function—a high-level cognitive function that requires conscious awareness—while participants watched a brief (8 min) and highly engaging movie by Alfred Hitchcock. The investigators found that the movie's executive demands, assessed quantitatively and qualitatively in independent control groups, predicted similar activity across individual participants in the frontal and parietal cortex, regions that support executive processing (25–29). Thus, the time-course of the fronto-parietal activation provided a template for decoding whether behaviorally non-responsive patients have similar cognitive experiences to healthy individuals in response to the executive demands of the movie. Using this approach, Naci and colleagues demonstrated that a patient who had been behaviorally non-responsive and thought to lack consciousness for 16 years was consciously aware and could continuously engage in complex thoughts about real-world events unfolding over time (7). Thus, they provided strong evidence that some patients who are entirely behaviorally non-responsive can retain conscious awareness of their external environment (17, 24).

However, *awareness of oneself*, an aspect of consciousness routinely tested for at the patient's bedside, is more elusive and harder to measure, even in patients who demonstrate awareness of their external environment, such as those thought to be in a minimally conscious state (30). Traditionally, awareness of oneself, or internal awareness, has been assessed through self-report and, as a result, it is challenging to measure in its complete absence. Therefore, the extent to which some DoC patients, especially those who remain entirely behaviorally non-responsive and are diagnosed to be in a VS, are capable of internal awareness remains unclear. In the healthy brain, the focus of conscious awareness is thought to switch naturally over time between its internal and external aspects (31–33), a relationship mediated by the fluctuating juxtaposition or anti-correlation of functional time-courses of the default mode network (DMN) and fronto-parietal networks, as observed in the resting state (34–36). Although recent studies suggest a role for the DMN in facilitating goal-oriented behavior (37–39), this network has been shown to support a variety of internally-driven processes, including autobiographical memory, imagination, thinking about the self (40–46), and internal awareness (31, 33). Furthermore, the DMN decreases in activity when attention is directed externally (34, 47, 48), but increases in response to introspectively-oriented cognitive processes (44, 46). By contrast, the networks extending in the frontal and parietal cortices, including the dorsal attention (DAN) and executive control (ECN) networks are thought to mediate externally-driven cognitive processes, including attention, inhibition and executive control, that support external awareness (49, 50). The fronto-parietal networks increase in activity when attention is directed to external stimuli in cognitive tasks (35, 36, 51, 52).

Thus, although the relationship between the DMN and DAN/ECN to one another may depend upon the paradigm employed and the goals of the subject (53, 54), the juxtaposition of their functional time-courses is critical for the naturally ongoing switches between internal and external awareness (31–33). As the DMN and fronto-parietal networks are juxtaposed to one another at rest (34–36), and dissociate further when attention is directed externally, we reasoned that their functional responses would be maximally juxtaposed to one another during a highly engaging stimulus.

In this study, we did not investigate internal awareness directly, but rather investigated the *potential for* internal as well as external awareness in DoC patients including patients in the vegetative and minimally conscious states. To this end, we asked whether the pattern of juxtaposition between the DMN and DAN/ECN functional time-courses observed in healthy controls (31, 55) was present in patients, who had previously demonstrated evidence of external awareness. To this end, we used the aforementioned highly engaging short movie by Alfred Hitchcock to drive the recruitment of the fronto-parietal networks and its maximal disengagement from the DMN relative to the resting state baseline, in a control group of healthy participants and severely brain-injured patients with disorders of consciousness. To circumvent the limitations of behavioral testing based on the clinical evaluation and ensure that patients categorized as unconscious indeed showed no wilful brain responses, each patient underwent a functional Magnetic Resonance Imaging (fMRI)-based assessment with a previously established command-following protocol for detecting covert awareness (5, 56). Initially, we investigated the functional connectivity of the DMN and DAN/ECN in the healthy controls during movie viewing relative to the baseline conditions. Subsequently, we tested whether DoC patients, who demonstrated independent covert external awareness, differently from patients who did not, showed a juxtaposition between the DMN and fronto-parietal functional time-courses that was strengthened by the complex stimulus.

## Methods

### Participants

Ethical approval was obtained from the Health Sciences Research Ethics Board and the Psychology Research Ethics Board of Western University, in London Canada. All healthy participants were right-handed, native English speakers and had no history of neurological disorders. They gave informed written consent and were remunerated for their time. Thirteen and twelve healthy volunteers participated in experiment 1 and 2, respectively. The data of healthy volunteers was previously reported in studies by Naci et al. (7, 18). A convenience sample of 18 DoC patients participated in experiment 3. The patients' respective substitute decision makers provided informed written consent. Three patients were excluded from final analyses. Of these, one was excluded because of large structural brain damage and extremely enlarged ventricles that would have rendered any further analysis impossible. A second patient was excluded due to excessive movement in the scanner, which caused the termination of the scanning session. The third patient was excluded due to a “locked-in syndrome” diagnosis. Patient 1 appeared twice in the data set, with the corresponding two different scanning visits 2 years apart. In visit 1, the patient showed the ability to perform the command following task in the scanner, whereas in visit 2 there was no evidence of command following. These differences may have been due to fluctuations in arousal or a genuine change in the patient's status of consciousness. Based on the results of the fMRI analysis, the patient's data from the 2 visits were treated as independent samples for the purpose of subsequence group analysis. Activity-based analyses on data from a subset of the patient cohort were previously reported in Naci et al. (7, 18) and Naci and Owen (5). Prior to commencing the scanning sessions, all patients were tested behaviorally at their bedside (outside of the scanner) with the Coma Recovery Scale-Revised (CRS-R) (57), which assessed each patient's behavioral responsivity along 6 sub-scales: auditory, visual, motor, oromotor/verbal, communication, and arousal (Table 1). All patients were clinically diagnosed as either VS or minimally conscious state [MCS; 30] at the time of the image acquisition based on the CRS-R. Table 2 provides an overview of the demographic and clinical information, as well as the results of the fMRI command-following protocol for each patient.

**Table 1:** Coma Recovery Scale—Revised subscale scores assessed prior to the fMRI session.

**Patient ID**	**Diagnosis**	**Auditory**	**Visual**	**Motor**	**Oromotor/verbal**	**Communication**	**Arousal**
1 Visit 1	VS	1 - Auditory startle	0 - None.	2 - Flexion withdrawal 2	1 - Oral reflexive 1	0 - None 0	2 - Eye opening without stimulation
Visit 2	VS	1 - Auditory startle	1 - Visual startle	2 - Flexion withdrawal	1 - Oral reflexive	0 - None	2 - Eye opening without stimulation
2	MCS	1 - Auditory startle	3 - Visual pursuit	2 - Flexion withdrawal	1 - Oral reflexive	0 - None	2 - Eye opening without stimulation
3	VS	1 - Auditory startle	1 - Visual startle	1 - Abnormal posturing	1 - Oral reflexive	0 - None	2 - Eye opening without stimulation
4	MCS	3 - Reproducible movement to command	3 - Visual pursuit	2 - Flexion withdrawal	2 -Vocaliza-tion/oral movement	0 - None	3 - Attention
5	MCS	1 - Auditory startle	3 - Visual pursuit	2 - Flexion withdrawal	1 - Oral reflexive	0 - None	1 - Eye opening with stimulation
6	VS	1 - Auditory startle	1 - Visual startle	2 - Flexion withdrawal	1 - Oral reflexive	0 - None	1 - Eye opening with stimulation
7	VS	0 - None	0 - None	2 - Flexion withdrawal	1 - Oral reflexive	0 - None	2 - Eye opening without stimulation
8	VS	2 - Localization to sound	1 - Visual startle	1 - Abnormal posturing	0 - None	0 - None	2 - Eye opening without stimulation
9	VS	0 - None	1 - Visual startle	0 - None	0 - None	0 - None	2 - Eye opening without stimulation
10	MCS	2 - Localization to sound	3 - Visual pursuit	1 - Abnormal posturing	1 - Oral reflexive	0 - None	2 - Eye opening without stimulation
11	MCS	4 - Consistent movement to command	4 - Object localization: reaching	4 - Automatic motor response	1 - Oral reflexive	1 - Non-functional: intentional	1 - Eye opening with stimulation
12	VS	1 - Auditory startle	0 - None	1 - Abnormal posturing	1 - Oral reflexive	0 - None	1 - Eye opening with stimulation
13	VS	1 - Auditory startle	0 - None	2 - Flexion withdrawal	1 - Oral reflexive	0 - None	1 - Eye opening with stimulation
14	VS	1 - Auditory startle	1 - Visual startle	0 - None	1 - Oral reflexive	0 - None	2 - Eye opening without stimulation
15	MCS	1 - Auditory startle	3 - Visual pursuit	1 - Abnormal posturing	1 - Oral reflexive	0 - None	1 - Eye opening with stimulation

*VS, vegetative state; MCS, minimally conscious state*.

**Table 2:** The patients' demographic and clinical information, fMRI command-following protocol results and functional connectivity results.

**Patient ID**	**Age range**	**Diagnosis**	**Interval since ictus, months**	**Score on CRS-R**	**Etiology**	**Command following (attention)**	**Movie DMN-DAN Connectivity**	**Resting DMN-DAN Connectivity**
1	Visit 1: 22–25	VS	67	6	TBI	+	0.21	0.32
	Visit 2: 26–30	VS	89	7		−	0.38	0.32
2	31–35	MCS	445	9	HBI	+	0.44	0.48
3	18–21	VS	68	6	HBI	−	0.43	0.31
4	26–30	MCS	36	13	HBI	+	0.35	0.37
5	46–50	MCS	234	8	HBI	+	0.19	0.24
6	56–60	VS	38	6	HBI	−	0.37	0.13
7	31–35	VS	25	5	HBI	−	0.47	0.35
8	18–21	VS	3	6	HBI	+	0.29	0.46
9	41–45	VS	248	3	TBI	+	0.33	0.45
10	22–25	MCS	69	9	TBI	+	0.62	0.64
11	46–50	MCS	148	15	TBI	−	0.60	0.54
12	51–55	VS	11	4	HBI	−	0.63	0.64
13	51–55	VS	79	5	HBI	−	0.60	0.52
14	18–21	VS	49	5	HBI	−	0.66	0.25
15	36–40	MCS	38	7	TBI	+	0.13	0.38

*VS, vegetative state; MCS, minimally conscious state; TBI, traumatic brain injury; HBI, hypoxic-ischemic brain injury; CRS-R, Coma Recovery Scale—Revised (57). In the “Command Following (Attention)” column, “+” denotes that the patient demonstrated a positive result, or showed evidence of command-following in the scanner; “–”denotes that the patient showed no evidence of command-following during this task. The Movie connectivity and Resting connectivity columns describe functional connectivity (as described by z-values) between the DMN and DAN*.

### Stimuli and design

In experiment 1, a group of healthy participants (*N* = 13) were scanned in two different conditions, resting state (8 min) and movie-viewing (8 min) in the same session. Participants were instructed to simply relax in the resting state, and to pay attention to the movie during the stimulation condition. The movie consisted of an edited version of Alfred Hitchcock's black-and-white movie “Bang! You're Dead.” It depicted a 5-year-old boy, who finds his uncle's revolver, partially loads it with bullets, and plays with it at home and in public, unaware of its power and danger. Sound in the scanner was delivered over scanner-compatible noise canceling headphones.

In experiment 2, healthy participants (*N* = 12) watched a visually and auditory scrambled version of the Hitchcock movie sequence inside the scanner. To create the scrambled condition, very brief (one second) audio-visual segments of the movie were pseudo-randomized, retaining the sensory properties (visual and auditory) while removing the narrative. Written feedback at the end of the scanning session confirmed that participants were not able to uncover a storyline in the scrambled movie, or relate it to stored knowledge of previous movies they had seen.

In experiment 3, DoC patients (*N* = 15) were scanned in the resting state and during viewing of the intact movie in the same session. The condition order was counterbalanced and the same procedure and scanning parameters were used for the patients as for healthy controls. To control for patient's wakefulness, eye opening was monitored inside the scanner with an infrared camera. Due to the highly limited time in the scanner, severely brain-injured patients did not undergo the scrambled movie baseline condition.

### Patient behavioral assessment and command-following fMRI paradigm

Awareness fluctuates greatly in the chronic DoC patient group, and therefore the behavioral diagnosis based on the gold-standard measure of CRS-R is likely to fluctuate (58). Furthermore, in some cases, as for CMD patients (59), awareness is difficult to spot behaviorally. Therefore, it was a priority of this manuscript to base evaluation of awareness on an assessment that would not falsely put covertly responding “VS patients” in a group of non-responders. Accordingly, to ensure an objective standard of awareness that was independent of the behavioral diagnosis, and also relevant but independent to the movie paradigm, awareness was assessed with a previously validated fMRI task (5) in the same scanning session as the movie paradigm.

To account for the variability of the behavioral diagnosis, we conducted on average 7 CRS-R assessments for each patient during the week of their research visit at our facility. Given this well-documented fluctuation, to follow as closely as possible the level of awareness that might be picked up by the fMRI task, here we reported the CRS-R score of the scanning day.

Each patient underwent a command following scan in the same scanning session. Stimuli. The stimuli were eleven single words (“one,” “two,” “three,” “four,” “five,” “six,” “seven,” “eight,” “nine,” “yes,” “no”). Design. The fMRI selective auditory attention paradigm has been previously described in healthy individuals (56) and patients with DoC (5), and is designed to identify the ability to follow commands to selectively attending to stimuli, by recruiting top-down attention. On each trial, participants were instructed to either count a target word (“yes” or “no”) presented among pseudorandom distractors (spoken digits one to nine), or to relax. Each trial had an on/off design: sound (~22.5 s) followed by silence (10 s). The scan lasted 5 min, including instructions.

As seen in Table 2, the results of the command following task were broadly consistent with the MCS patients' clinical diagnosis that indicated overt awareness—all but one MCS patient, who fell asleep in the scanner, were able to perform the selective attention task in the scanner [see (60, 61) for diverging results on MCS patients who could not perform a command-following task in the MRI scanner]. By contrast, 3 out of 10 VS patients showed positive command following results. This is consistent with previous findings showing that a proportion of patients clinically diagnosed as VS are nevertheless able to modulate brain activity to command (2, 3, 15). The results of this command-following task were used to split the patient cohort into two groups: DoC+ patients were able to perform the command-following task in the scanner, DoC– patients were not able to do so. Further analyses were then performed independently for these two groups.

### Functional data acquisition

All participants were scanned in a 3 Tesla Siemens Tim Trio MRI scanner at the Robarts Research Institute in London, Canada. A 32-channel head coil was used for functional and anatomical scans. We acquired functional images during movie viewing (246 scans) and resting-state (256 scans) by a T2^*^-weighted echo-planar sequence [33 slices, voxel size = 3 × 3 × 3 mm^3^, interslice gap = 25%, repetition time = 2,000 ms, echo time (TE) = 30 ms, matrix size = 64 × 64, flip angle (FA) = 75 degrees]. Furthermore, a T1-weighted 3D magnetization prepared rapid acquisition gradient echo (MPRAGE) sequence was used for anatomical scans [voxel size = 1 × 1 × 1 mm^3^, TE = 4.25 ms, matrix size = 240 × 256 × 192, FA = 9 degrees]. The total anatomical scanning time was 5min 38sec. All scanning parameters were the same for healthy participants and patients.

### Preprocessing

For preprocessing and data analysis we used SPM8 (Wellcome Institute of Cognitive Neurology, http://www.fil.ion.ucl.ac.uk/spm/software/spm8/) and the AA pipeline software [(62), www.automaticanalysis.org]. All preprocessing and data analysis steps were the same for healthy participants and patients. We discarded the first five volumes of each run to avoid T1-saturation effects. The preprocessing procedure included slice-time correction, motion correction, normalization into Montreal Neurological Institute (MNI) space and spatial smoothing with a Gaussian kernel of 10mm full width at half maximum. Furthermore, we applied a temporal high-pass filter with a cut-off of 1/128 Hz to each voxel and regressed out the six motion parameters (x, y, z, roll, pitch, yaw). To investigate any confounding effects of movement differences between groups and conditions, we additionally calculated the mean frame-wise displacement (63) for each participant and compared them using a mixed ANOVA, as well as paired *t*-tests. Healthy participants did not differ significantly in movement, as assessed by frame-wise displacement values, between the movie viewing and resting state condition [*t*_(12)_ = −1.91, *p* = 0.08]. Similarly, the patients' movement did not differ significantly. A two-factor mixed ANOVA on motion, with factors group (DoC+, DoC–) and condition (movie, rest) showed no significant main effects [group: *F*_(1)_ = 0.28, *p* = 0.60; condition: *F*_(1)_ = 1.01, *p* = 0.33] and no significant interaction [*F*_(1, 1)_ = 0.53, *p* = 0.48].To avoid the formation of artificial anti-correlations, a confounding effect previously reported by Murphy et al. (64) and Anderson et al. (65), we performed no global signal regression.

### Functional network definition

**Figure 1:**
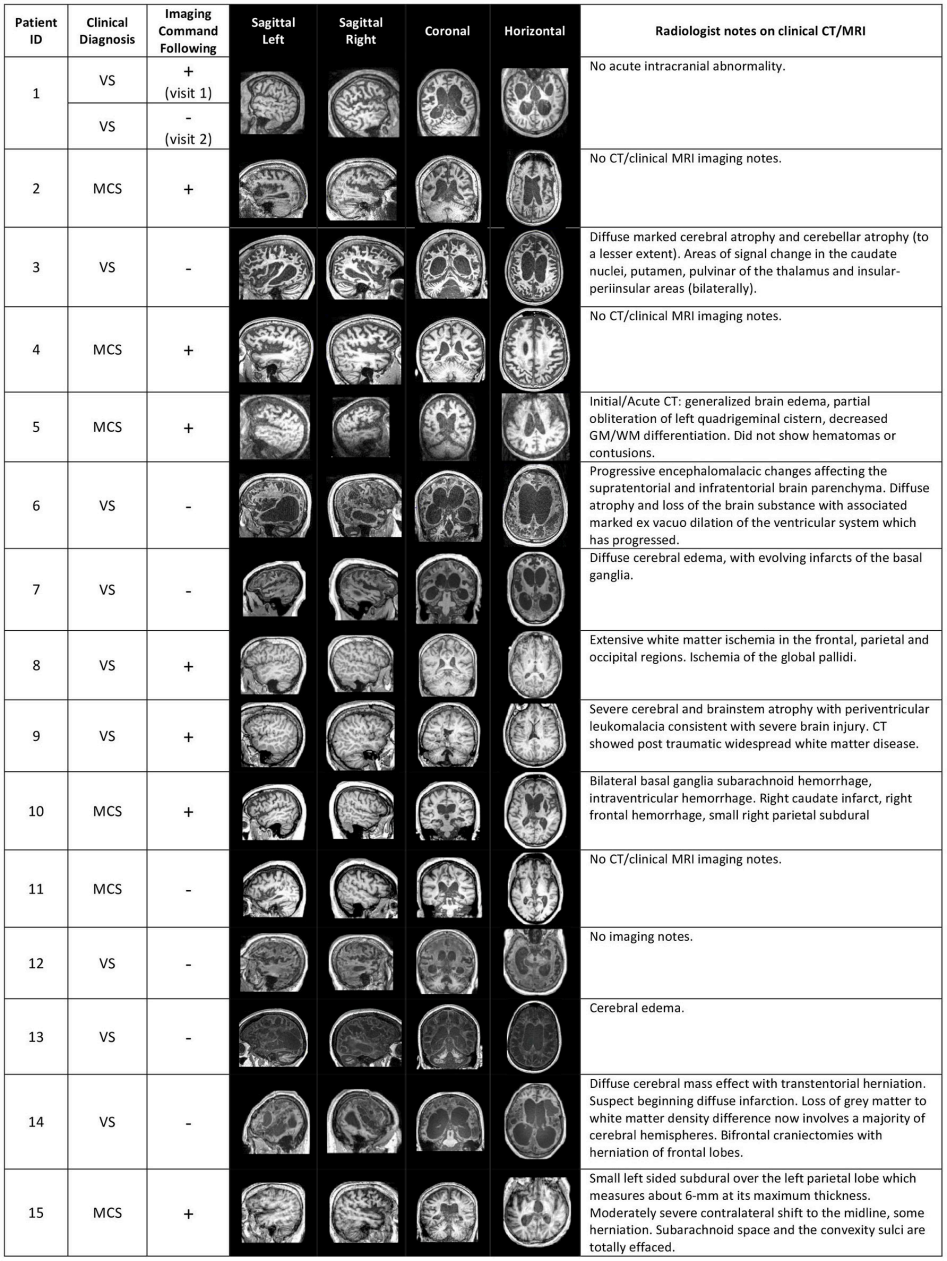
Structural brain information for the patient cohort. Columns 4–7 give an overview of structural MRI images that were taken for each patient. On the right, clinical notes by radiologists on previous computerized tomography (CT) and/or MRI scans are listed. The sign “+” or “–” in the “Imaging Command Following” column describes whether the patient was able to successfully complete a command-following task in the MRI scanner (+), or not (−).

We analyzed functional connectivity within and between the three key networks that are involved in higher order processes: the DMN, DAN and ECN. The functional networks were defined based on functionally specific regions of interest (ROIs) (19 in total, 10mm spheres), from well-established landmark coordinates published in Raichle (66). MNI coordinates for ROIs of each network can be found in Table 3. For the analysis of functional connectivity based on a set of network nodes pre-defined in the healthy literature (MNI standard neurological space), each patient's brain was normalized to the healthy template. Some of the ROIs may not be optimally located in a subset of the patients due to the varying location and extent of damage (see Figure 1 for an overview of structural information on the patients' brains). The mechanism of functional re-organization that follows brain injury and leads to loss of consciousness in some cases, whereas in others to preservation of consciousness, remains poorly understood and is the focus of active research (59, 67) outside of the scope of this manuscript. Therefore, although it is impossible to ascertain the structure-to-preserved function mapping for each individual patient, we expected that any damage within the regions of interest in each patient's brain would add noise to the brain activity measurement and reduce the power to detect an effect. Therefore, if results in brain-injured patients confirmed a-priory hypotheses based on the healthy control group, they would likely present a conservative estimate of the underlying effect.

**Table 3:** Overview of the regions of interests for the DMN, DAN, and ECN.

**Network**	**ROI**	**MNI coordinates**		
Default mode network	Posterior cingulate/precuneus	0	−52	27
	Medial prefrontal	−1	54	27
	Left lateral parietal	−46	−66	30
	Right lateral parietal	49	−63	33
	Left inferior temporal	−61	−24	−9
	Right inferior temporal	58	−24	−9
Dorsal attention network	Left frontal eye field	−29	−9	54
	Right frontal eye field	29	−9	54
	Left posterior IPS	−26	−66	48
	Right posterior IPS	26	−66	48
	Left anterior IPS	−44	−39	45
	Right anterior IPS	41	−39	45
	Left MT	−50	−66	−6
	Right MT	53	−63	−6
Executive control network	Dorsal medial PFC	0	24	46
	Left anterior PFC	−44	45	0
	Right anterior PFC	44	45	0
	Left superior parietal	−50	−51	45
	Right superior parietal	50	−51	45

*IPS, Intraparietal sulcus; MT, Middle temporal area; PFC, Prefrontal cortex*.

### Functional connectivity analysis

**Figure 2:**
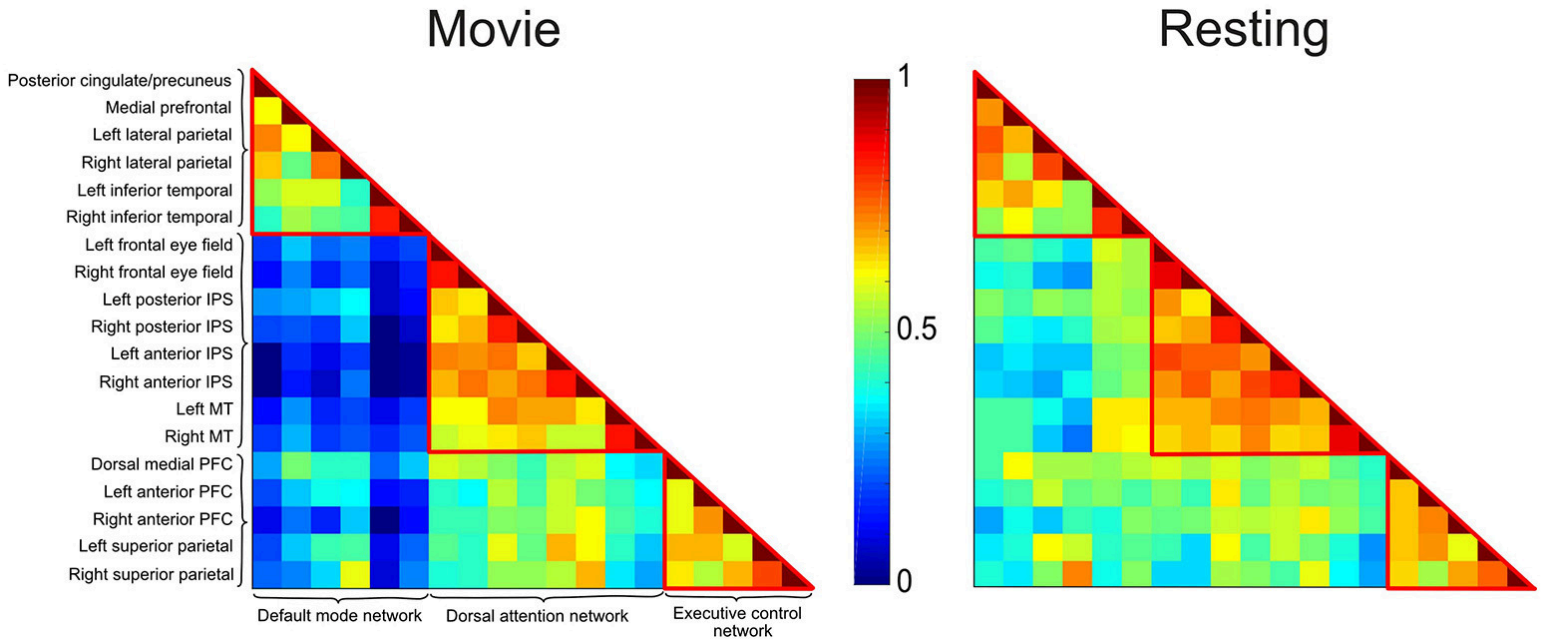
Correlation matrices depicting the DMN, DAN, and ECN during movie viewing and resting state conditions in healthy participants. Each cell represents the color-coded connectivity between an ROI to itself (middle diagonal) or another region. The labels for the ROIs in each network are displayed on the left-hand side. Warm/cool colors depict high/low correlations as per the heat bar in the middle of the graph. During movie viewing, healthy participants showed decreased functional connectivity between the DMN and the DAN/ECN as compared to the resting state. Functional connectivity within each network, and between the DAN and ECN did not differ significantly between the two conditions.

The preprocessed mean BOLD time series of each ROI was extracted and correlated (Pearson correlation) with the time courses of all the other ROIs. We note that Pearson correlation is a basic FC measure that, while it does not directly imply causal relations between neural regions, is advantageous for its minimal assumptions regarding the true nature of brain interactions and breath of its use in the neuroscientific literature, and thus fitting to the aims of this investigation. Based on these Pearson's correlations, we created a 19 × 19 correlation matrix (Figure 2). We performed this procedure separately for the movie and resting state for each participant. The average over all ROIs within a network was computed and a two-way repeated measures ANOVA and Bonferroni-corrected pairwise comparisons were performed to evaluate effects of interest. To account for the non-normalized distribution of correlation values (68), all statistical analyses were performed on z-transformed correlation values, using Fisher's r-to-z transformation. For visualization purposes, we re-transformed these *z*-values in correlation values.

## Results

### Healthy participants

**Figure 3:**
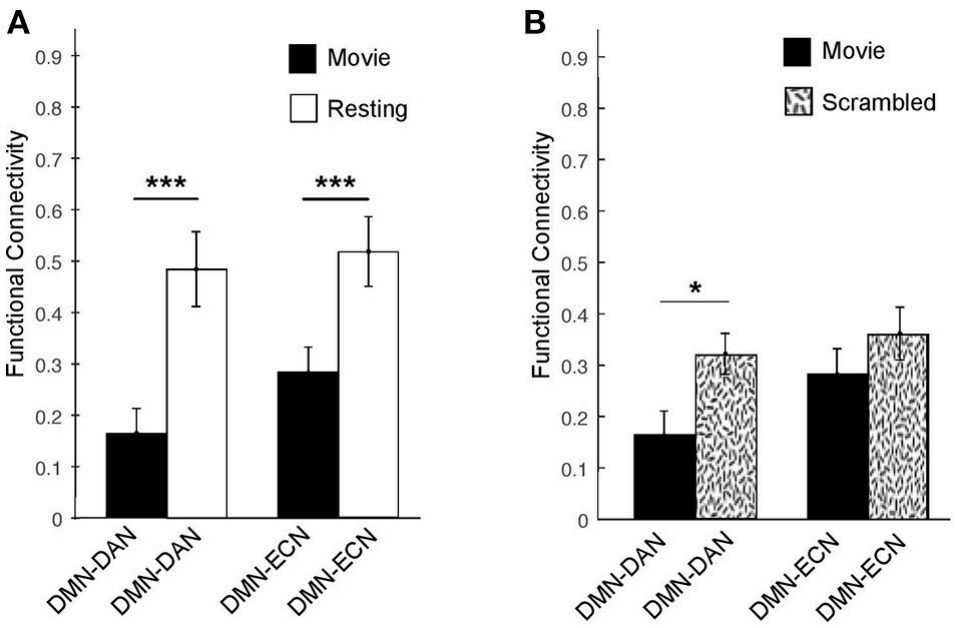
Averaged functional connectivity between the DMN and DAN/ECN during the movie and baseline conditions in healthy participants. **(A)** During movie viewing, the DMN–DAN [*t*_(12)_ = 4.58, *p* < 0.001] and the DMN–ECN [*t*_(12)_ = 4.03, *p* < 0.005] connectivity was significantly down-regulated in healthy participants relative to the resting state. **(B)** The DMN–DAN [*t*_(11)_ = −2.289, *p* < 0.05], but not the DMN–ECN, connectivity was significantly down-regulated during the intact movie relative to its scrambled version, suggesting that the connectivity down-regulation was driven by the movie's higher-order properties including its narrative. *p < 0.05, **p < 0.01, ***p < 0.005.

The correlation matrices for the movie viewing and resting state conditions, including all ROIs for the three networks, are shown in Figure 2. A two-way repeated measures ANOVA with factors condition (movie, rest) and connectivity (*within*-networks, *between*-networks) revealed a significant interaction effect [*F*_(1, 1)_ = 18.52, *p* < 0.001]. During movie viewing the connectivity between the DMN and DAN [*t*_(12)_ = 4.58, *p* < 0.001] and DMN and ECN [*t*_(12)_ = 4.03, *p* < 0.005] were significantly down-regulated during the movie relative to the resting state (Figure 3A). As the measure of connectivity (Pearson correlation) reflected the degree of similarity between the networks' functional time-courses, this result demonstrated that the functional response of each of the DAN/ECN became more dissimilar to that of the DMN during the movie relative to the resting state baseline. By comparison, functional connectivity between DAN and ECN [*t*_(12)_ = 0.23, *p* = 0.82], as well as functional connectivity within the functional networks [DMN: *t*_(12)_ = 2.15, *p* = 0.052; DAN: *t*_(12)_ = 1.35, *p* = 0.20; ECN: *t*_(12)_ = 0.75, *p* = 0.47] did not differ between the movie and resting state condition.

To further investigate whether this dissociation was indeed related to the processing of the movie's higher-order properties, including its narrative, or merely driven by the presence of sensory stimulation in the movie relative to the resting state condition, we investigated the connectivity between these networks during the intact movie relative to its scrambled version, which retained the sensory features but was devoid of the narrative (Figure 3B). Relative to the scrambled movie, in the intact movie we found significant down-regulation of the DMN-DAN connectivity [*t*_(11)_ = −2.289, *p* < 0.05], but not the DMN–ECN connectivity (Figure 3B). This suggested that the modulation of the DMN–DAN, but not DMN–ECN, connectivity during movie viewing reflected the processing of the movie's higher-order features, including its narrative. This result was consistent with a recent study showing heightened functional differentiation with increasing stimuli meaningfulness (69).

### DoC patients

The results in the healthy controls suggested that the heightened differentiation between the DMN and DAN aspect of the fronto-parietal network was driven by the movie's high-order properties including its narrative. Subsequently, we investigated whether behaviorally non-responsive patients who retained covert external awareness (labeled here DoC+), and those who showed no such evidence (labeled here DoC–) [see command following (attention) in Table 2], showed heightened differentiation of the functional response of the DMN and DAN networks in response to movie viewing relative to their resting state baseline connectivity.

**Figure 4:**
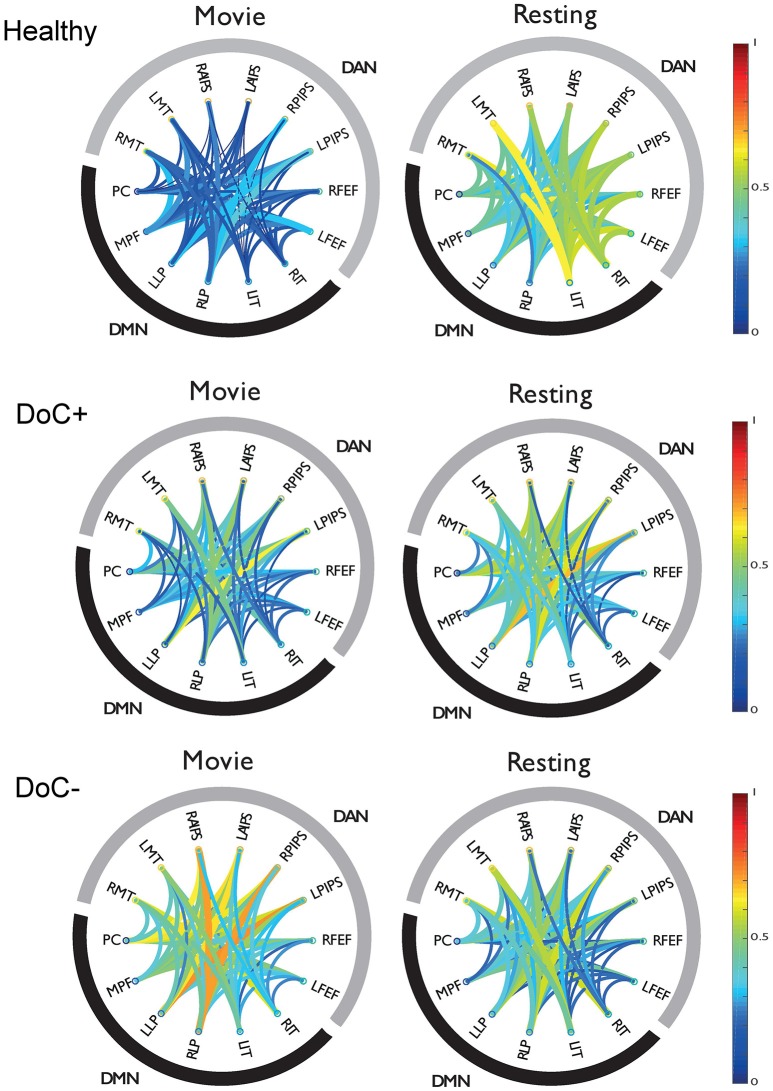
DMN-DAN connectivity in healthy participants and DoC patients. The schema-balls provide an overview of individual ROI connectivity and depict all possible between-ROI connections for the DMN and DAN. Warm/cool colors depict high/low correlations as per the heat bar to the right of the graph. Healthy participants showed a down-regulation of DMN–DAN connectivity during movie viewing relative to the resting state. Similarly, the DoC+ patients showed a down-regulation of DMN–DAN connectivity during movie viewing [*t*_(7)_ = −3.31, *p* < 0.05]. By contrast, DoC-patients did not show this effect. RMT, right middle temporal visual area; LMT, left middle temporal visual area; RAIPS, right anterior intraparietal sulcus; LAIPS, left anterior intraparietal sulcus; RPIPS, right posterior intraparietal sulcus; LPIPS, left posterior intraparietal sulcus; RFEF, right frontal eye field; LFEF, left frontal eye field; RIT, right inferior temporal; LIT, left inferior temporal; RLP, right lateral parietal; LLP, left lateral parietal; MPF, medial prefrontal; PC, posterior cingulate/precuneus.

DoC+ patients showed a significant down-regulation of DMN–DAN connectivity, suggesting heightened differentiation of the networks' functional response during the movie viewing relative to the resting state (Figure 4) [*t*_(7)_ = −3.31, *p* < 0.05]. By contrast, DoC- patients showed no down-regulation of the DMN–DAN connectivity, but rather a significant up-regulation of this connectivity [*t*_(7)_ = 2.99, *p* < 0.05] during the movie relative to the resting state.

**Figure 5:**
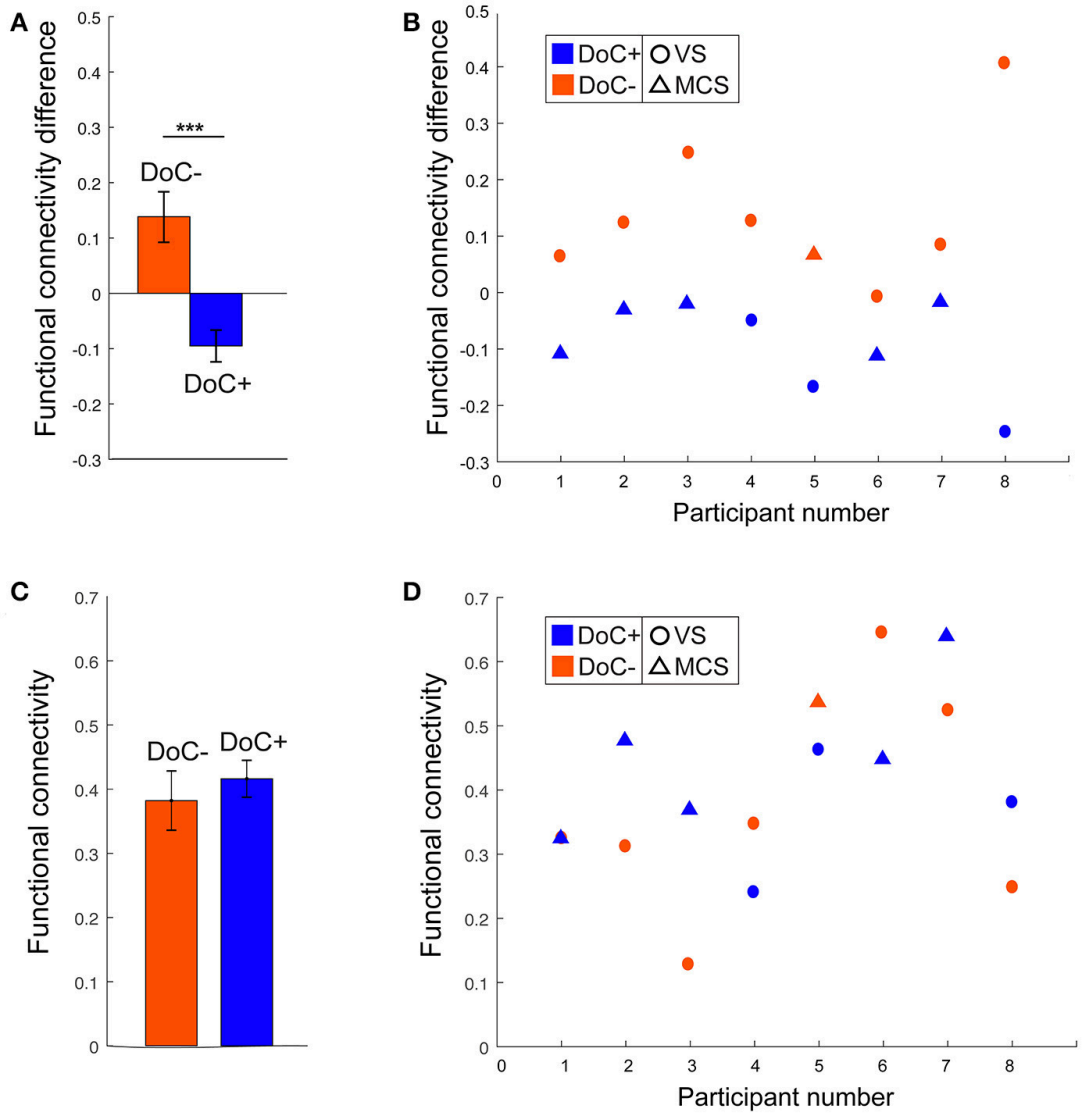
DMN–DAN connectivity difference between movie viewing and the resting state and during resting state only in DoC patients. **(A)** The DoC+ and DoC– groups differed significantly on their DMN–DAN connectivity modulation during movie viewing relative to resting state [*t*_(15)_ = 4.23, *p* < 0.005]. **(B)** Visual inspection suggested that individual DoC+ patients displayed a down-regulation of DMN–DAN connectivity during movie viewing, illustrated by a negative value for the difference between movie and resting DMN–DAN connectivity. **(C,D)** During the resting state, there was no significant difference in DMN–DAN connectivity between the DoC+ and DoC– groups **(C)**, and no such difference could be observed at the individual patient level **(D)**. ***p < 0.005.

The modulatory effect of movie viewing on the DMN–DAN connectivity was highly significant different between the two patient groups [*t*_(15)_ = 4.23, *p* = 0.001; Figure 5A]. Moreover, the down-regulation of DMN–DAN connectivity in the DoC+ group, and the opposite effect in the DoC– group was visible in individual patient (Figure 5B), although, we caution that the current analysis is not optimized to investigate statistical significance at the single-subject level. By contrast, the DMN–DAN connectivity during resting state did not differentiate the two patient types (Figures 5C,D).

## Discussion

In this study, we asked whether patients with disorders of consciousness retain the potential for internal as well as external conscious awareness. To address this question, we investigated the juxtaposed relationship between the DMN and fronto-parietal (DAN, ECN) networks' functional time-courses, which are thought to support the naturally occurring fluctuations and dominance switching between internal and external awareness (31–33) in healthy individuals. We used a highly engaging external stimulus—a short movie by Alfred Hitchcock to drive maximal juxtaposition of the DMN and fronto-parietal networks relative to the resting state and scrambled, meaningless movie baseline conditions. Initially we investigated the connectivity between the DMN and fronto-parietal networks in response to movie viewing and baseline conditions in healthy controls, and subsequently, in two groups of severely brain-injured patients with DoC, one comprised of patients who demonstrated independent neuroimaging-based evidence of covert external awareness, and the other of those who did not.

Healthy controls showed significantly heightened differentiation between the DMN and the DAN in response to movie viewing relative to their resting state and scrambled movie time-courses. This result suggested that the heightened differentiation during movie viewing was driven by the movie's higher-order features, including its narrative. This finding was consistent with previous studies showing a heightened dissociation between the default mode and fronto-parietal networks on paradigms requiring externally-directed attention [(48, 52, 55), but see (70) for diverging results]. The connectivity between the DMN and ECN was not modulated by the movie's higher-order features, which may be explained by differential engagement by the movie paradigm of the DAN and ECN. Movie viewing required the ability to orient and sustain attention to the incoming auditory input, and discriminate the saliency and contextual relevance of the sensory inputs with respect to the evolving narrative—a function subserved primarily by the DAN (50), and it did not require behavioral response planning or monitoring— a function subserved primarily by the ECN (49).

Similar to healthy controls, severely brain-injured patients who had demonstrated independent evidence of covert external awareness showed significantly enhanced differentiation between the DMN and DAN functional time-course in response to movie viewing relative to the resting state baseline. This result suggested the presence of functional integrity in the default mode and fronto-parietal networks and their relationship to one another. It is worth noting that we did not investigate internal awareness directly, but rather the potential for internal awareness in addition to external awareness as indicated by the pattern of juxtaposed time-courses of these networks. Similar to the effect in healthy participants, these networks became more strongly juxtaposed to one another in response to movie viewing, relative to their resting baseline, suggesting the *potential* for internal as well as external awareness during complex stimulus processing.

By contrast, this effect was not present in chronic DoC patients who showed no fMRI-based evidence of covert awareness. Conversely, these patients showed a diminished differentiation between the DMN and DAN time-courses during the movie viewing relative to the resting state baseline. This was consistent with previous studies showing a loss of functional differentiation between brain networks in loss of consciousness (71–73). The loss of functional differentiation in response to movie viewing relative to the resting state suggests that, in severely brain-injured patients who likely lack conscious awareness, the stimulus-evoked feed-forward processing cascade is echoed undifferentiated throughout the brain leading to similar functional responses across different networks. In this study, we had no differential hypotheses for the MCS+ vs. MCS– patients and, on this basis, we did not differentiate between MCS patients. Further, the small number of MCS patients (*N* = 6) did not facilitate further separation into groups. However, it would be interesting to investigate in future studies differences between MCS+ and MCS– patients in this regard, and we would hypothesize that MCS+ patients would be more likely than MCS– to show the heighted differentiation between the DMN and DAN networks during the movie paradigm relative to the resting state, which the current data suggest is indicative of the potential for internal awareness during viewing of a naturalistic narrative.

Visual inspection suggested that this effect was present at the single-subject level and differentiated patients who had covert awareness from those who did not, not only at the group but also at the individual level and thus, may be sufficiently robust to facilitate detection of covert awareness in individual patients in the absence of other neuroimaging assessments. This hypothesis requires formal testing with a large number of patients in future studies. Notably, this effect was not present when looking at DMN–DAN connectivity in the resting state alone, suggesting that richly evocative stimulation is appropriate for differentiating between behaviorally non-responsive patients who retain covert awareness and those who don't, based on the integrity of consciousness supporting networks. Previous studies have suggested several benefits of the movie-viewing paradigm over the resting state for investigating the functional integrity of brain networks in healthy individuals. Foremost, movie-viewing creates an immersive experience that naturally engages attention, rendering participants less likely to fall asleep and less liable to arousal fluctuations present in the resting state condition (74), it reduces movement (75, 19), and leads to strong brain activity (21, 22). Importantly, a complex and plot-driven naturalistic stimulus engages functionally distinct brain systems in a stereotypical way across different individuals (7, 17, 18, 20, 21, 23) that enables comparisons between different participants with minimized inter-subject variance with respect to specific perceptual and cognitive processes. Furthermore, the test-retest reliability of functional connectivity analyses during movie-viewing has been shown superior relative to the resting state, on average by 50% (76).

Our results suggest that these benefits extend to patient populations. To date, a large number of clinical studies investigating functional connectivity have focused on the resting state condition, due to its ease of acquisition in patient populations [e.g., 77–83] and have provided significant insights on functional disruption in various populations relative to neurologically typical individuals. However, this approach cannot account for an important characteristic of healthy neural processing, that is, the brain's ability to re-organize its connectivity in response to external stimulation. Here, we show that naturalistic paradigms, which present complex real-world information evolving over time, can dissociate between groups of severely brain-injured chronic DoC patients with and without covert awareness, an approach that may also yield important insights when extended to other patient populations (84). Particularly, the ease of patient engagement with reduced movement and reduced arousal fluctuations is beneficial for testing a range of patient populations that, similarly to brain-injured patients, exhibit large fluctuations in arousal, impaired motor control, and compromised attention span, and thus, are difficult to test with conventional paradigms that target cognition.

## Author contributions

AH and LN: conceptualization; LN, AH, and RC: methodology; LG-L: patient recruitment; LN: data acquisition; AH and LN: formal analyses; AH and LN: manuscript preparation; BS and AO: feedback; LN: supervision.

### Conflict of interest statement

The authors declare that the research was conducted in the absence of any commercial or financial relationships that could be construed as a potential conflict of interest. The reviewer MM declared a past co-authorship with one of the authors AO to the handling Editor.

## References

[1] Owen AM Coleman MR Boly M Davis MH Laureys S Pickard JD. Detecting awareness in the vegetative state. Science (2006) 313:1402. 10.1126/science.1130197 16959998

[2] Monti MM Vanhaudenhuyse A Coleman MR Boly M Pickard JD Tshibanda L. Willful modulation of brain activity in disorders of consciousness. N Engl J Med. (2010) 362:579–89. 10.1056/NEJMoa0905370 20130250

[3] Cruse D Chennu S Chatelle C Bekinschtein TA Fernández-Espejo D Pickard JD. Bedside detection of awareness in the vegetative state: a cohort study. Lancet (2012) 378:2088–94. 10.1016/S0140-6736(11)61224-5 22078855

[4] Bardin JC Fins JJ Katz DI Hersh J Heier LA Tabelow K. Dissociations between behavioural and functional magnetic resonance imaging-based evaluations of cognitive function after brain injury. Brain (2011) 134:769–82. 10.1093/brain/awr005 21354974 PMC3044833

[5] Naci L Owen AM. Making every word count for nonresponsive patients. JAMA Neurol. (2013) 70:1235–41. 10.1001/jamaneurol.2013.3686 23939634

[6] Fernández-Espejo D Owen AM. Detecting awareness after severe brain injury. Nat Rev Neurosci. (2013) 14:801–9. 10.1038/nrn3608 24088810

[7] Naci L Cusack R Anello M Owen AM. A common neural code for similar conscious experiences in different individuals. Proc Natl Acad Sci USA (2014) 111:14277–82. 10.1073/pnas.1407007111 25225384 PMC4191782

[8] Bodien YG Giacino JT Edlow BL. Functional MRI motor imagery tasks to detect command following in traumatic disorders of consciousness. Front Neurol. (2017) 8:688. 10.3389/fneur.2017.00688 29326648 PMC5741595

[9] PPlum F Posner JB The Diagnosis of Stupor and Coma, Vol. 19 Oxford: Oxford University Press (1982).

[10] Laureys S Owen AM Schiff ND. Brain function in coma, vegetative state, and related disorders. The Lancet Neurology, (2004) 3, 537–546. 10.1016/S1474-4422(04)00852-X 15324722

[11] Owen AM. Disorders of consciousness. Ann N Y Acad Sci. (2008) 1124:225–38. 10.1196/annals.1440.013 18400933

[12] The Multi-Society Task Force on PVS. Medical aspects of the persistent vegetative state. N Engl J Med. (1994) 330:1499–508. 10.1056/NEJM199405263302107 7818633

[13] Laureys S Celesia GG Cohadon F Lavrijsen J León-Carrión J Sannita WG. Unresponsive wakefulness syndrome: a new name for the vegetative state or apallic syndrome. BMC Med. (2010) 8:68. 10.1186/1741-7015-8-68 21040571 PMC2987895

[14] Schnakers C Vanhaudenhuyse A Giacino J Ventura M Boly M Majerus S. Diagnostic accuracy of the vegetative and minimally conscious state: clinical consensus versus standardized neurobehavioral assessment. BMC Neurol. (2009) 9:35. 10.1186/1471-2377-9-35 19622138 PMC2718857

[15] Kondziella D Friberg CK Frokjaer VG Fabricius M Møller K. Preserved consciousness in vegetative and minimal conscious states: systematic review and meta-analysis. J Neurol Neurosurg Psychiatry (2015) 87:485–92. 10.1136/jnnp-2015-310958 26139551

[16] Schiff ND. Cognitive motor dissociation following severe brain injuries. JAMA Neurol. (2015) 72:1413–5. 10.1001/jamaneurol.2015.2899 26502348

[17] Sinai L Owen AM Naci L. Mapping preserved real-world cognition in severely brain-injured patients. Front Biosci (Landmark Ed). (2017) 22:815–23. 10.2741/4518 27814648

[18] Naci L Sinai L Owen AM. Detecting and interpreting conscious experiences in behaviorally non-responsive patients. NeuroImage (2017) 145(Pt B):304–13. 10.1016/j.neuroimage.2015.11.059 26679327

[19] Centeno M Tierney TM Perani S Shamshiri EA StPier K Wilkinson C. Optimising EEG-fMRI for localisation of focal epilepsy in children. PLoS ONE (2016) 11:e0149048. 10.1371/journal.pone.0149048 26872220 PMC4752259

[20] Hasson U Nir Y Levy I Fuhrmann G Malach R. Intersubject synchronization of cortical activity during natural vision. Science (2004) 303:1634–40. 10.1126/science.1089506 15016991

[21] Bartels A Zeki S. Brain dynamics during natural viewing conditions—a new guide for mapping connectivity in vivo. Neuroimage (2005) 24:339–49. 10.1016/j.neuroimage.2004.08.044 15627577

[22] Bartels A Zeki S Logothetis NK. Natural vision reveals regional specialization to local motion and to contrast-invariant, global flow in the human brain. Cereb Cortex (2007) 18:705–17. 10.1093/cercor/bhm107 17615246

[23] Hasson U Malach R Heeger DJ. Reliability of cortical activity during natural stimulation. Trends Cogn Sci. (2010) 14:40–8. 10.1016/j.tics.2009.10.011 20004608 PMC2818432

[24] Naci L Graham M Owen AM Weijer C. Covert narrative capacity: mental life in patients thought to lack consciousness. Ann Clin Transl Neurol. (2017) 4:61–70. 10.1002/acn3.376 28078316 PMC5221458

[25] Sauseng P Klimesch W Schabus M Doppelmayr M. Fronto-parietal EEG coherence in theta and upper alpha reflect central executive functions of working memory. Int J Psychophysiol. (2005) 57:97–103. 10.1016/j.ijpsycho.2005.03.018 15967528

[26] Hampshire A Owen AM. Fractionating attentional control using event-related fMRI. Cereb Cortex (2006) 16:1679–89. 10.1093/cercor/bhj116 16436686

[27] Duncan J. The multiple-demand (MD) system of the primate brain: mental programs for intelligent behaviour. Trends Cogn Sci. (2010) 14:172–9. 10.1016/j.tics.2010.01.004 20171926

[28] Ptak R Schnider A. The attention network of the human brain: relating structural damage associated with spatial neglect to functional imaging correlates of spatial attention. Neuropsychologia (2011) 49:3063–70. 10.1016/j.neuropsychologia.2011.07.008 21787795

[29] Barbey AK Colom R Solomon J Krueger F Forbes C Grafman J. An integrative architecture for general intelligence and executive function revealed by lesion mapping. Brain (2012) 135:1154–64. 10.1093/brain/aws021 22396393 PMC3326251

[30] Giacino JT Ashwal S Childs N Cranford R Jennett B Katz DI. The minimally conscious state definition and diagnostic criteria. Neurology (2002) 58:349–53. 10.1212/WNL.58.3.349 11839831

[31] Vanhaudenhuyse A Demertzi A Schabus M Noirhomme Q Bredart S Boly M. Two distinct neuronal networks mediate the awareness of environment and of self. J Cogn Neurosci. (2011) 23:570–8. 10.1162/jocn.2010.21488 20515407

[32] Heine L Soddu A Gómez F Vanhaudenhuyse A Tshibanda L Thonnard M. Resting state networks and consciousness: alterations of multiple resting state network connectivity in physiological, pharmacological, and pathological consciousness states. Front Psychol. (2012) 3:195. 10.3389/fpsyg.2012.00295 22969735 PMC3427917

[33] Demertzi A Soddu A Laureys S. Consciousness supporting networks. Curr Opin Neurobiol. (2013) 23:239–44. 10.1016/j.conb.2012.12.003 23273731

[34] Greicius MD Krasnow B Reiss AL Menon V. Functional connectivity in the resting brain: a network analysis of the default mode hypothesis. Proc Natl Acad Sci USA. (2003) 100:253–8. 10.1073/pnas.0135058100 12506194 PMC140943

[35] Fox MD Snyder AZ Vincent JL Corbetta M Van Essen DC Raichle ME. The human brain is intrinsically organized into dynamic, anticorrelated functional networks. Proc Natl Acad Sci USA. (2005) 102:9673–8. 10.1073/pnas.0504136102 15976020 PMC1157105

[36] Sridharan D Levitin DJ Menon V. A critical role for the right fronto-insular cortex in switching between central-executive and default-mode networks. Proc Natl Acad Sci USA. (2008) 105:12569–74. 10.1073/pnas.0800005105 18723676 PMC2527952

[37] Spreng RN DuPre E Selarka D Garcia J Gojkovic S Mildner J. Goal-congruent default network activity facilitates cognitive control. J Neurosci. (2014) 34:14108–14. 10.1523/JNEUROSCI.2815-14.2014 25319706 PMC4198547

[38] Vatansever D Menon DK Manktelow AE Sahakian BJ Stamatakis EA. Default mode dynamics for global functional integration. J Neurosci. (2015) 35:15254–62. 10.1523/JNEUROSCI.2135-15.2015 26586814 PMC4649001

[39] Vatansever D Menon DK Stamatakis EA. Default mode contributions to automated information processing. Proc Natl Acad Sci USA. (2017) 114:12821–6. 10.1073/pnas.1710521114 29078345 PMC5715758

[40] Gusnard DA Akbudak E Shulman GL Raichle ME. Medial prefrontal cortex and self-referential mental activity: relation to a default mode of brain function. Proc Natl Acad Sci USA. (2001) 98:4259–64. 10.1073/pnas.071043098 11259662 PMC31213

[41] Wicker B Ruby P Royet JP Fonlupt P A relation between rest and the self in the brain? Brain Res Rev. (2003) 43:224–30. 10.1016/j.brainresrev.2003.08.003 14572916

[42] D'argembeau A Collette F Van der Linden M Laureys S Del Fiore G Degueldre C. Self-referential reflective activity and its relationship with rest: a PET study. Neuroimage (2005) 25:616–24. 10.1016/j.neuroimage.2004.11.048 15784441

[43] Beer JS. The default self: feeling good or being right? Trends Cogn Sci. (2007) 11:187–9. 10.1016/j.tics.2007.02.004 17347027

[44] Buckner RL Andrews-Hanna JR Schacter DL. The brain's default network. Ann N Y Acad Sci. (2008) 1124:1–38. 10.1196/annals.1440.011 18400922

[45] Schneider F Bermpohl F Heinzel A Rotte M Walter M Tempelmann C. The resting brain and our self: self-relatedness modulates resting state neural activity in cortical midline structures. Neuroscience (2008) 157:120–31. 10.1016/j.neuroscience.2008.08.014 18793699

[46] Andrews-Hanna JR Reidler JS Sepulcre J Poulin R Buckner RL. Functional-anatomic fractionation of the brain's default network. Neuron (2010) 65:550–62. 10.1016/j.neuron.2010.02.005 20188659 PMC2848443

[47] Raichle ME MacLeod AM Snyder AZ Powers WJ Gusnard DA Shulman GL. A default mode of brain function. Proc Natl Acad Sci USA. (2001) 98:676–82. 10.1073/pnas.98.2.676 11209064 PMC14647

[48] Shulman GL Fiez JA Corbetta M Buckner RL Miezin FM Raichle ME. Common blood flow changes across visual tasks: II. Decreases in cerebral cortex. J Cogn Neurosci. (1997) 9:648–63. 10.1162/jocn.1997.9.5.648 23965122

[49] Kroger JK Sabb FW Fales CL Bookheimer SY Cohen MS Holyoak KJ. Recruitment of anterior dorsolateral prefrontal cortex in human reasoning: a parametric study of relational complexity. Cereb Cortex (2002) 12:477–85. 10.1093/cercor/12.5.477 11950765

[50] Corbetta M Shulman GL. Control of goal-directed and stimulus-driven attention in the brain. Nat Rev Neurosci. (2002) 3:201–15. 10.1038/nrn755 11994752

[51] Seeley WW Menon V Schatzberg AF Keller J Glover GH Kenna H. Dissociable intrinsic connectivity networks for salience processing and executive control. J Neurosci. (2007) 27:2349–56. 10.1523/JNEUROSCI.5587-06.2007 17329432 PMC2680293

[52] Dosenbach NU Fair DA Miezin FM Cohen AL Wenger KK Dosenbach RA. Distinct brain networks for adaptive and stable task control in humans. Proc Natl Acad Sci USA. (2007) 104:11073–8. 10.1073/pnas.0704320104 17576922 PMC1904171

[53] Spreng RN Stevens WD Chamberlain JP Gilmore AW Schacter DL. Default network activity, coupled with the frontoparietal control network, supports goal-directed cognition. Neuroimage (2010) 53:303–17. 10.1016/j.neuroimage.2010.06.016 20600998 PMC2914129

[54] Smallwood J Brown K Baird B Schooler JW. Cooperation between the default mode network and the frontal–parietal network in the production of an internal train of thought. Brain Res. (2012) 1428:60–70. 10.1016/j.brainres.2011.03.072 21466793

[55] Kelly AC Uddin LQ Biswal BB Castellanos FX Milham MP. Competition between functional brain networks mediates behavioral variability. Neuroimage (2008) 39:527–537. 10.1016/j.neuroimage.2007.08.008 17919929

[56] Naci L Cusack R Jia VZ Owen AM. The brain's silent messenger: using selective attention to decode human thought for brain-based communication. J Neurosci. (2013) 33:9385–93. 10.1523/JNEUROSCI.5577-12.2013 23719806 PMC6618571

[57] Giacino JT Kalmar K Whyte J. The JFK coma recovery scale-revised: measurement characteristics and diagnostic utility. Arch Phys Med Rehabil. (2004) 85:2020–29. 10.1016/j.apmr.2004.02.033 15605342

[58] Wannez S Heine L Thonnard M Gosseries O Laureys S Coma Science Group Collaborators. The repetition of behavioral assessments in diagnosis of disorders of consciousness. Ann Neurol. (2017) 81:883–9. 10.1002/ana.24962 28543735

[59] Schiff ND. Altered consciousness. In: Winn R, editor. Youmans and Winn's Neurological Surgery, 7th Edn. New York, NY: Elsevier Saunders (2016). p. 203–8.

[60] Monti MM Coleman MR Owen AM Executive functions in the absence of behavior: functional imaging of the minimally conscious state. Prog Brain Res. (2009) 117:249–60. 10.1016/S0079-6123(09)17717-8 19818906

[61] Monti MM Rosenberg M Finoia P Kamau E Pickard JD Owen AM Thalamo-frontal connectivity mediates top-down cognitive functions in disorders of consciousness. Neurology (2015) 85:1–7. 10.1212/WNL.0000000000001123 PMC4336082 25480912

[62] Cusack R Vicente-Grabovetsky A Mitchell DJ Wild CJ Auer T Linke AC. Automatic analysis (aa): efficient neuroimaging workflows and parallel processing using Matlab and XML. Front Neuroinform. (2015) 8:90. 10.3389/fninf.2014.00090 25642185 PMC4295539

[63] Power JD Barnes KA Snyder AZ Schlaggar BL Petersen SE. Spurious but systematic correlations in functional connectivity MRI networks arise from subject motion. Neuroimage (2012) 59:2142–54. 10.1016/j.neuroimage.2011.10.018 22019881 PMC3254728

[64] Murphy K Birn RM Handwerker DA Jones TB Bandettini PA. The impact of global signal regression on resting state correlations: are anti-correlated networks introduced? Neuroimage (2009) 44:893–905. 10.1016/j.neuroimage.2008.09.036 18976716 PMC2750906

[65] Anderson JS Druzgal TJ Lopez-Larson M Jeong EK Desai K Yurgelun-Todd D. Network anticorrelations, global regression, and phase-shifted soft tissue correction. Hum Brain Mapp. (2011) 32:919–34. 10.1002/hbm.21079 20533557 PMC3220164

[66] Raichle ME. The restless brain. Brain Connect. (2011) 1:3–12. 10.1089/brain.2011.0019 22432951 PMC3621343

[67] Fernández-Espejo D Rossit S Owen AM. A thalamocortical mechanism for the absence of overt motor behavior in covertly aware patients. JAMA Neurol. (2015) 72:1442–50. 10.1001/jamaneurol.2015.2614 26501399

[68] Fisher RA Frequency distribution of the values of the correlation coefficient in samples from an indefinitely large population. Biometrika (1915) 10:507–21. 10.2307/2331838

[69] Boly M Sasai S Gosseries O Oizumi M Casali A Massimini M. Stimulus set meaningfulness and neurophysiological differentiation: a functional magnetic resonance imaging study. PLoS ONE (2015) 10:e0125337. 10.1371/journal.pone.0125337 25970444 PMC4430458

[70] Elton A Gao W. Task-positive functional connectivity of the default mode network transcends task domain. J Cogn Neurosci. (2015) 27:2369–81. 10.1162/jocn_a_00859 26244722

[71] Stamatakis EA Adapa RM Absalom AR Menon DK. Changes in resting neural connectivity during propofol sedation. PLoS ONE (2010) 5:e14224. 10.1371/journal.pone.0014224 21151992 PMC2996305

[72] Massimini M Ferrarelli F Sarasso S Tononi G. Cortical mechanisms of loss of consciousness: insight from TMS/EEG studies. Arch Ital Biol. (2012) 150:44–55. 10.4449/aib.v150i2.1361 23165870

[73] Casali AG Gosseries O Rosanova M Boly M Sarasso S Casali KR. A theoretically based index of consciousness independent of sensory processing and behavior. Sci Transl Med. (2013) 5:198ra105. 10.1126/scitranslmed.3006294 23946194

[74] Tagliazucchi E Laufs H. Decoding wakefulness levels from typical fMRI resting-state data reveals reliable drifts between wakefulness and sleep. Neuron (2014) 82:695–708. 10.1016/j.neuron.2014.03.020 24811386

[75] Vanderwal T Kelly C Eilbott J Mayes LC Castellanos FX. (2015). Inscapes: a movie paradigm to improve compliance in functional magnetic resonance imaging. NeuroImage 122:222–32. 10.1016/j.neuroimage.2015.07.069 26241683 PMC4618190

[76] Wang J Ren Y Hu X Nguyen VT Guo L Han J. Test–retest reliability of functional connectivity networks during naturalistic fMRI paradigms. Hum Brain Mapp. (2017) 38:2226–41. 10.1002/hbm.23517 28094464 PMC6867176

[77] Sorg C Riedl V Mühlau M Calhoun VD Eichele T Läer L. Selective changes of resting-state networks in individuals at risk for Alzheimer's disease. Proc Natl Acad Sci USA. (2007) 104:18760–5. 10.1073/pnas.0708803104 18003904 PMC2141850

[78] Greicius M. Resting-state functional connectivity in neuropsychiatric disorders. Curr Opin Neurol. (2008) 21:424–30. 10.1097/WCO.0b013e328306f2c5 18607202

[79] Boly M Tshibanda L Vanhaudenhuyse A Noirhomme Q Schnakers C Ledoux D. Functional connectivity in the default network during resting state is preserved in a vegetative but not in a braindead patient. Hum Brain Mapp. (2009) 30:2393–400. 10.1002/hbm.20672 19350563 PMC6870763

[80] Rosazza C Minati L. Resting-state brain networks: literature review and clinical applications. Neurol Sci. (2011) 32:773–85. 10.1007/s10072-011-0636-y 21667095

[81] Soddu A Vanhaudenhuyse A Demertzi A Bruno MA Tshibanda L Di H. Resting state activity in patients with disorders of consciousness. Funct Neurol. (2011) 26:37. 21693087 PMC3814510

[82] Lee MH Smyser CD Shimony JS. Resting-state fMRI: a review of methods and clinical applications. Amer J Neuroradiol. (2013) 34:1866–72. 10.3174/ajnr.A3263 22936095 PMC4035703

[83] Hannawi Y Lindquist MA Caffo BS Sair HI Stevens RD. Resting brain activity in disorders of consciousness A systematic review and meta-analysis. Neurology (2015) 84:1272–80. 10.1212/WNL.0000000000001404 25713001 PMC4366089

[84] Hasson U Avidan G Gelbard H Vallines I Harel M Minshew N. Shared and idiosyncratic cortical activation patterns in autism revealed under continuous real-life viewing conditions. Autism Res. (2009) 2:220–31. 10.1002/aur.89 19708061 PMC2775929

